# USING BIOLOGICAL AGING TO BETTER UNDERSTAND THE DIFFERENTIAL RETURNS TO EDUCATION AMONG BLACK AND WHITE OLDER ADULTS

**DOI:** 10.1093/geroni/igae098.1222

**Published:** 2024-12-31

**Authors:** Mateo Farina

**Affiliations:** University of Texas at Austin, Austin, Texas, United States

## Abstract

Numerous studies have documented “differential health returns” for education among older Black and White adults in the United States. Despite being a more select group, Black older adults with higher levels of education see a smaller return in health risk declines than White older adults. This pattern has been observed for cardiovascular and metabolic conditions, disability, and mortality. Underlying several of these age-related health conditions may be accelerated biological aging, which may provide further insight into why education returns differ across many health outcomes. In this study, we use the Health and Retirement 2016 Venous Blood Study to examine the differences in the education gradient for Black and White older adults for accelerated aging as defined by the newly developed Expanded Biological Age measure. We use a series of linear regression models to examine the association of education with accelerated biological aging and we examine whether the differences can be partly explained by health behaviors, experiences of discrimination, or psychosocial stress. Preliminary findings show that, compared to Black older adults White older adults have a larger gradient (greater returns) to education when evaluating accelerated biological aging: 3.2-year difference between those without a high school diploma and a bachelor’s degree for Blacks, and a 5.3-year difference for Whites. Health behaviors had no impact on the differences across groups, but we find some indication that stress and discrimination may have a role in mitigating the health benefits for well-educated older black adults.

